# Effects of Thai Local Ingredient Odorants, *Litsea cubeba* and Garlic Essential Oils, on Brainwaves and Moods

**DOI:** 10.3390/molecules26102939

**Published:** 2021-05-15

**Authors:** Apsorn Sattayakhom, Sumethee Songsamoe, Gorawit Yusakul, Kosin Kalarat, Narumol Matan, Phanit Koomhin

**Affiliations:** 1 School of Allied Health Sciences, Walailak University, Nakhonsithammarat 80160, Thailand; apsorn.sa@wu.ac.th; 2 Center of Excellence in Innovation on Essential Oil, Walailak University, Nakhonsithammarat 80160, Thailand; sumethee.so@wu.ac.th (S.S.); nnarumol@wu.ac.th (N.M.); 3 School of Agricultural Technology, Walailak University, Nakhonsithammarat 80160, Thailand; 4 School of Pharmacy, Walailak University, Nakhonsithammarat 80160, Thailand; gorawit.yu@wu.ac.th; 5 School of Informatics, Walailak University, Nakhonsithammarat 80160, Thailand; kosin.ka@wu.ac.th; 6 School of Medicine, Walailak University, Nakhonsithammarat 80160, Thailand

**Keywords:** *Litsea cubeba* oil, garlic oil, turmeric oil, electroencephalography

## Abstract

The functional food market is growing with a compound annual growth rate of 7.9%. Thai food recipes use several kinds of herbs. Lemongrass, garlic, and turmeric are ingredients used in Thai curry paste. Essential oils released in the preparation step create the flavor and fragrance of the famous tom yum and massaman dishes. While the biological activities of these ingredients have been investigated, including the antioxidant, anti-inflammatory, and antimicrobial activities, there is still a lack of understanding regarding the responses to the essential oils of these plants. To investigate the effects of essential oil inhalation on the brain and mood responses, electroencephalography was carried out during the non-task resting state, and self-assessment of the mood state was performed. The essential oils were prepared in several dilutions in the range of the supra-threshold level. The results show that *Litsea cubeba* oil inhalation showed a sedative effect, observed from alpha and beta wave power reductions. The frontal and temporal regions of the brain were involved in the wave alterations. Garlic oil increased the alpha wave power at lower concentrations; however, a sedative effect was also observed at higher concentrations. Lower dilution oil induced changes in the fast alpha activity in the frontal region. The alpha and beta wave powers were decreased with higher dilution oils, particularly in the temporal, parietal, and occipital regions. Both *Litsea cubeba* and turmeric oils resulted in better positive moods than garlic oil. Garlic oil caused more negative moods than the others. The psychophysiological activities and the related brain functions require further investigation. The knowledge obtained from this study may be used to design functional food products.

## 1. Introduction

The size of the functional food market was approximately 161.49 billion in 2018, and its compound annual growth rate has been 7.9% since 2014. Thai foods are rich in medicinal ingredients [1]. Traditional Thai curry paste has been used for centuries as part of many famous foods in Thai restaurants. Tom yum and massaman are renowned among these lists. Over the centuries, Thai curry paste recipes were developed, and these vary by region. The composition is comprised of local Thai herbs, which include lemongrass, garlic, turmeric, fragrant basils, kapi (shrimp paste), lime juice, sugar, and chilies. Sugar is more often added in the central region of Thailand. Turmeric is also added in the southern region [2]. There are several studies that suggest biological properties of these ingredients. Lemongrass fragrance showed antioxidant properties using a 2,2-diphenyl-1-picryl-hydrazyl-hydrate scavenging test [3]. Antimicrobial and anti-inflammatory properties have been tested. The effects of lemongrass essential oil related to the nervous system functions were investigated. Oil injections reduced nociceptive face-rubbing behavior in an animal study [4]. The anxiolytic-like and antidepressant activities in animal study were also reported for lemongrass essential oil treatment [5]. Garlic also shows antioxidant, antimicrobial, and anti-inflammatory properties [6,7]. Garlic oil and its components showed antidepressant-like effects via the increase of serotonin and the dopamine level [8]. *Curcuma longa* essential oil showed antioxidant activity and anti-inflammatory activity in a macrophage and animal model, respectively. For the nervous system, turmeric essential oil showed antinociceptive activity in acetic acid-induced writhing movements in mice [9]. The anxiolytic-like effect of curcumin was also investigated in an animal study [10]. Lime juice is an important source of antioxidants, such as flavonoids and vitamin C. This juice also confers favorable physicochemical characteristics of refreshing and a pleasing flavor to products [11].

Fragrances or odorants have effects on human psychophysiology. The perception of fragrances is enabled by odorants and odorant receptors in the olfactory system. Aromatherapy is an alternative medicine using fragrances in treatments to heal a person’s ailments, purportedly, in terms of mind, body, and soul [12]. Each essential oil is comprised of a mixture of components on different levels, which influences the flavor and aroma. The *Litsea cubeba* aroma is similar to lemongrass with more citrus notes. Turmeric and garlic are widely used as an ingredient in traditional cooking due to their unique aromas. The unique aroma of turmeric is dependent on a combination of ar-turmerone and β-turmerone with α-curcumene, β-sesquiphellandrene, and other kinds of alcohols. The garlic aroma is from the sulfur-based substances—thiosulfinates and their degradation products [13]. *Litsea cubeba*, garlic, and *Curcuma longa* oils share components with several types of essential-oil-bearing plants. Limonene is found in *Litsea cubeba*, lemongrass, bergamot, caraway, eucalyptus, lemon, etc. Phellandrene is found in *Curcuma longa*, rosemary, eucalyptus, etc. Essential oil affects the human nervous system depending on its composition and concentration [14]. Inhalation of essential oil has been reported to alter the arousal state of the human brain ranging from stimulatory effects to sedative effects. Alterations of psychophysiological and brain functions in response to odors have been found in the medicinal and cosmetic industries. There is no evidence regarding *Litsea cubeba*, garlic, and *Curcuma longa* essential oil inhalations on human brain responses; therefore, in this study, we investigated the effects of essential oil inhalation on human brain responses and moods. We hypothesized the stimulating effect of garlic oil and sedative effect of *Litsea cubeba* oil base on the increase in dopamine and serotonin levels and γ-aminobutyric acid receptor inhibition, respectively.

## 2. Results

### 2.1. Gas Chromatography–Mass Spectrometry (GC–MS) Analyses of Litsea Cubeba and Garlic Oils

The GC–MS analysis of the *Litsea cubeba* oil identified the compounds presented in Table 1. The major compounds were α-citral (33.36%), β-citral (26.78%), and dl-limonene (20.44%). The GC–MS analysis of the garlic oil identified the compounds presented in Table 2. The major compounds were diallyl sulfide (30.17%), diallyl disulphide (24.40%), and diallyl trisulfide (18.00%). The GC–MS analysis of the *Curcuma longa* oil showed ar-tumerone, tumerone, β-turmerone, α-terpinene, and α-phellandrene as the main components (Table S1).

### 2.2. Effects of Essential Oil Inhalation on Brainwaves and Moods

The essential oil dilutions used in this study were in the range of the supra-threshold level. The subjects of this study were university students aged around 21 years old. From our previous study, female subjects respond to odors better than male subjects [15]. Therefore, we recruited only females in this study. All the volunteers had a normal smell capability. *Litsea cubeba* oil shared similar components with lemongrass oil, such as citral, limonene, β-myrcene, geraniol, and citronellol. In this study, we chose *Litsea cubeba* oil due to its industrial-scale readiness.

**Figure 1:**
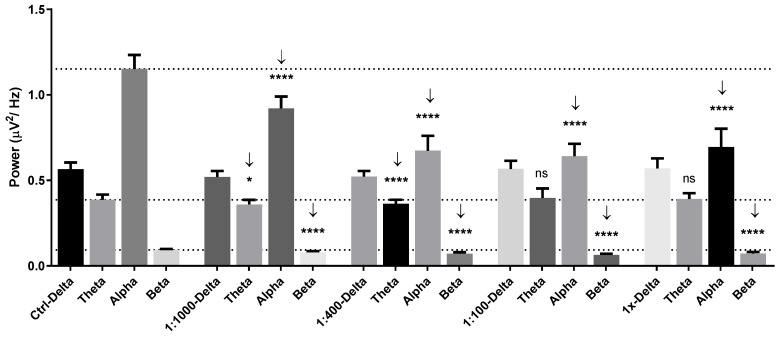
Power spectral analysis of human brainwaves characterized by frequencies consisting of delta, theta, alpha, and beta wave powers in control, 1:1000 dilution, 1:400 dilution, 1:100 dilution, and undiluted groups of *Litsea cubeba* oil. Not significant (ns), *, and **** represent no statistical differences, *p* < 0.05, and *p* < 0.0001, respectively.

*Litsea cubeba* oil inhalation produced a reduction in brain wave powers at the 1:1,000, 1:400, 1:100, and undiluted dilutions. For lower concentrations—the 1:1,000 and 1:400 dilutions—theta, alpha, and beta wave powers were reduced compared with the control. The alpha and beta wave powers were decreased with the 1:100 and undiluted dilution (Figure 1). For the 1:1000 dilution, the slow alpha wave power was especially decreased in the frontal region of the brain. The beta wave power in the range of low beta and mid beta wave power was decreased during *Litsea cubeba* oil inhalation.

**Figure 2:**
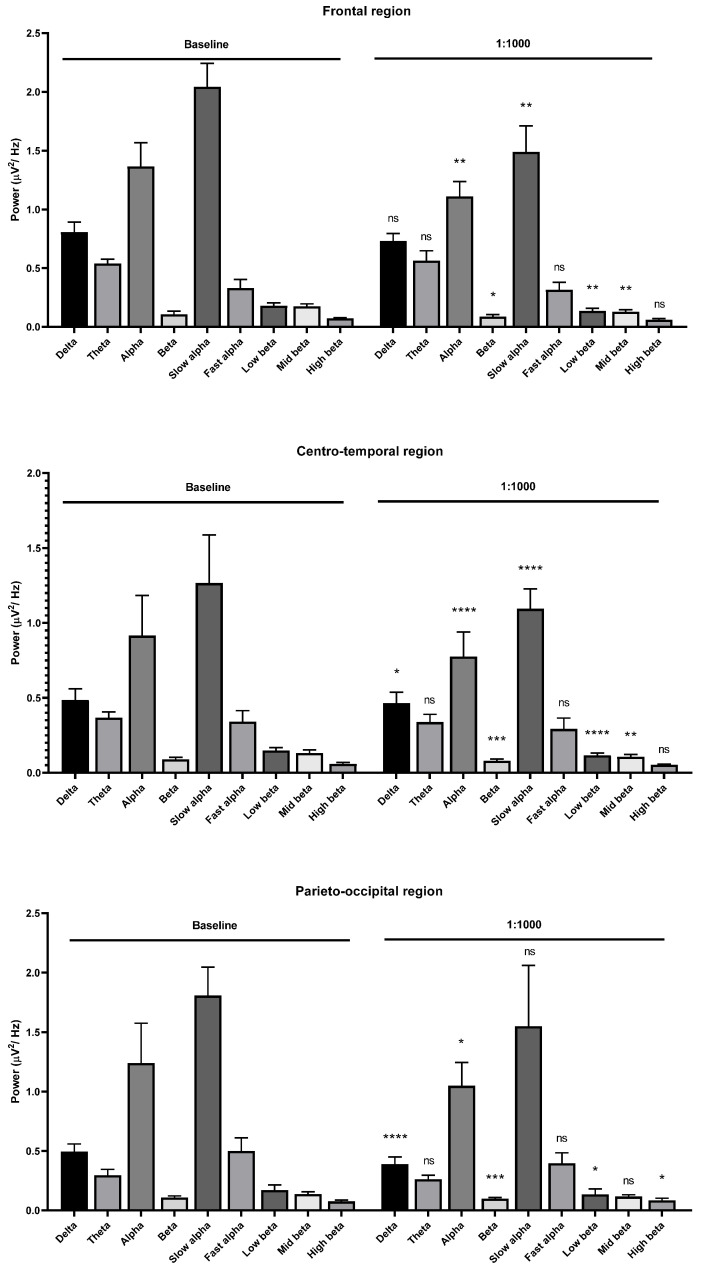
Effects of *Litsea cubeba* oil inhalation at a 1:1000 dilution on the delta, theta, alpha, beta, slow alpha, fast alpha, low beta, mid beta, and high beta wave powers: (**upper**) frontal region; (**middle**) centro-temporal region; (**lower**) parieto-occipital region. *p* value: not significant (ns) ≥ 0.05, * < 0.05, ** < 0.01, *** < 0.001, and **** < 0.0001.

**Figure 3:**
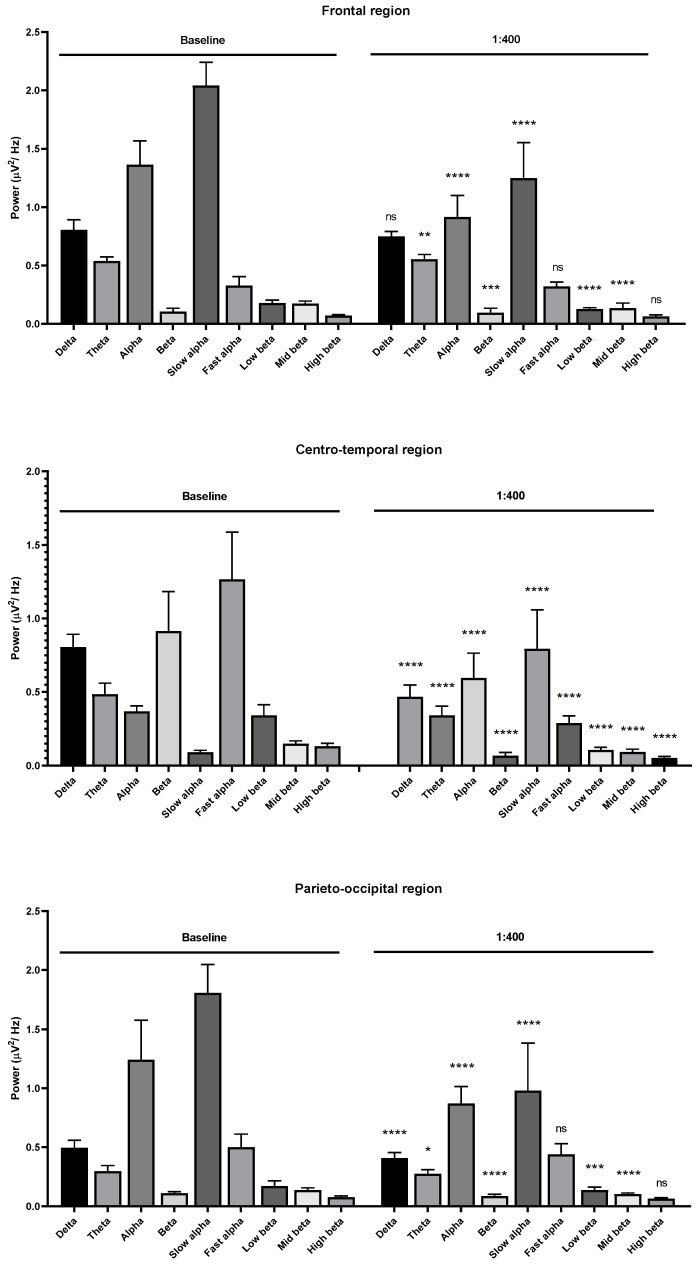
Effects of *Litsea cubeba* oil inhalation at a 1:400 dilution on the delta, theta, alpha, beta, slow alpha, fast alpha, low beta, mid beta, and high beta wave powers: (**upper**) frontal region; (**middle**) centro-temporal region; (**lower**) parieto-occipital region. *p* value: not significant (ns) ≥ 0.05, * < 0.05, ** < 0.01, *** < 0.001, and **** < 0.0001.

Centro-temporal region alteration was observed in the same fashion as in the frontal region. Slight changes of beta wave power were observed in the occipital region compared with the frontal and centro-temporal region for 1:1000 dilution (Figure 2). Beta wave and alpha wave powers were clearly decreased in the frontal, centro-temporal, and parieto-occipital regions for the 1:400 oil dilution. The slow alpha, low beta, and mid beta wave powers were decreased in the frontal and parieto-occipital regions. The slow alpha, fast alpha, theta, low beta, mid beta, and high beta wave powers were decreased in the centro-temporal region during oil inhalation (Figure 3).

**Figure 4:**
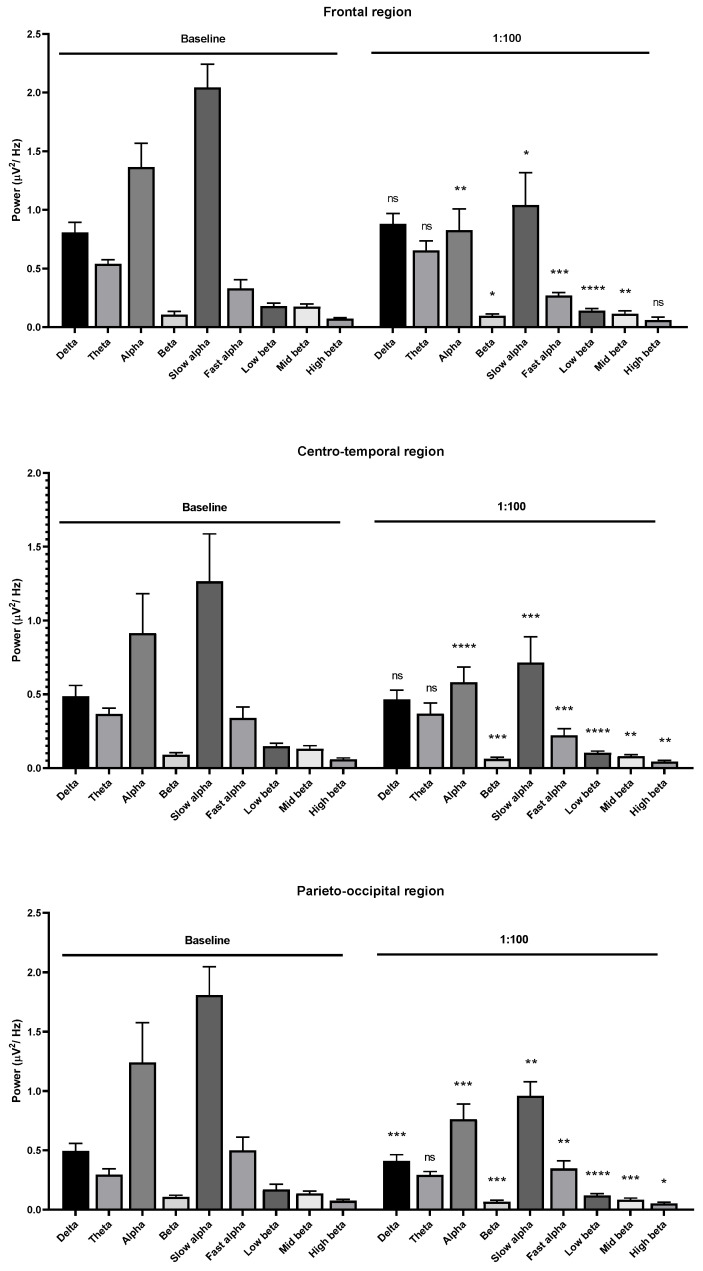
Effects of *Litsea cubeba* oil inhalation at a 1:100 dilution on the delta, theta, alpha, beta, slow alpha, fast alpha, low beta, mid beta, and high beta wave powers: (**upper**) frontal region; (**middle**) centro-temporal region; (**lower**) parieto-occipital region. *p* value: not significant (ns) ≥ 0.05, * < 0.05, ** < 0.01, *** < 0.001, and **** < 0.0001.

**Figure 5:**
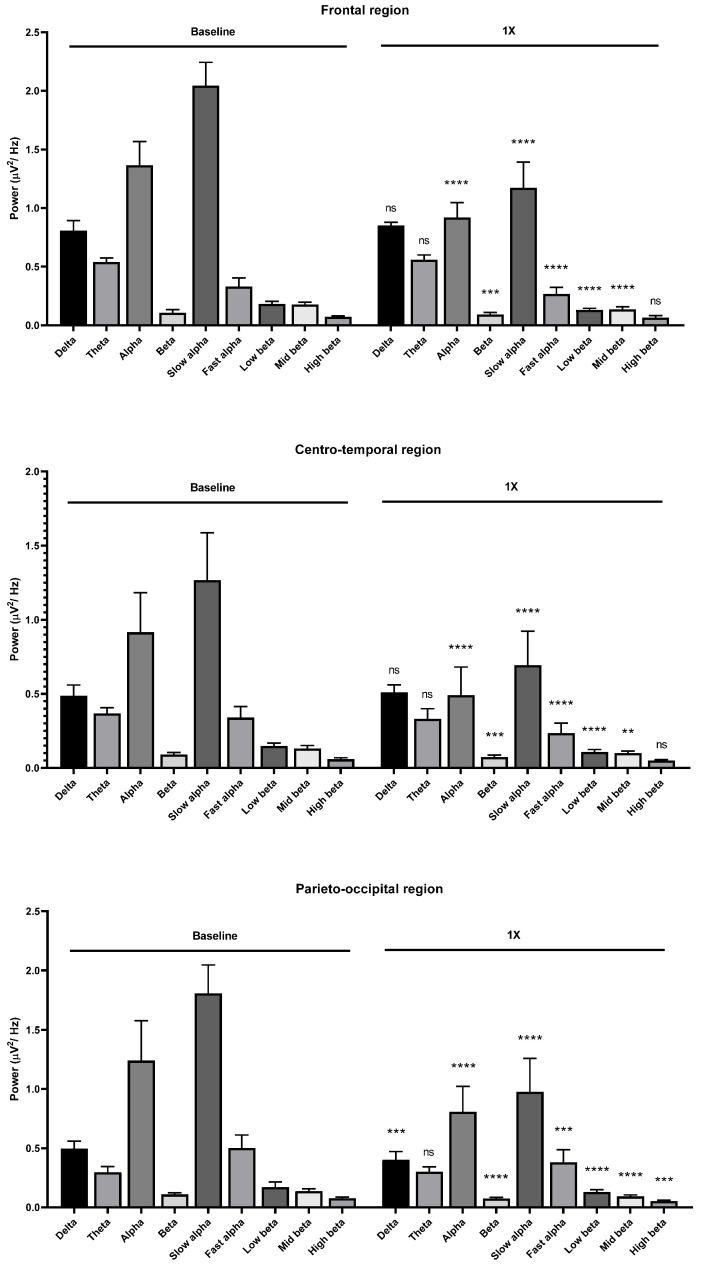
Effects of *Litsea cubeba* oil inhalation of an undiluted dilution (1X) on the delta, theta, alpha, beta, slow alpha, fast alpha, low beta, mid beta, and high beta wave powers: (**upper**) frontal region; (**middle**) centro-temporal region; (**lower**) parieto-occipital region. *p* value: not significant (ns) ≥ 0.05, ** < 0.01, *** < 0.001, and **** < 0.0001.

**Figure 6:**
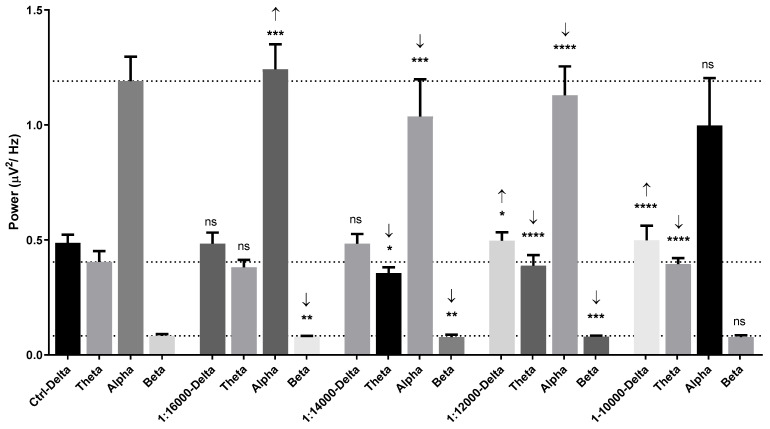
Power spectral analysis of human brainwaves characterized by frequencies consisting of delta, theta, alpha, and beta wave powers in control, 1:1000 dilution, 1:400 dilution, 1:100 dilution, and undiluted garlic oil groups. Not significant (ns), *, **, *** and **** represent no statistical differences, *p* < 0.05, and *p* < 0.01, *p* < 0.001, and *p* < 0.0001, respectively.

For the 1:100 and undiluted dilutions, the results showed a reduction in both alpha and beta wave powers among frontal, centro-temporal, parieto-occipital regions (Figure 4 and Figure 5). All the results of *Litsea cubeba* oil inhalations suggested a sedative effect of this oil inhalation. Due to the low threshold of garlic oil, we chose 1:16,000, 1:14,000, 1:12,000, and 1:10,000 dilutions as the supra-threshold concentrations. The results showed that alpha wave power increased with the 1:16,000 dilution but the beta wave power decreased (Figure 6).

**Figure 7:**
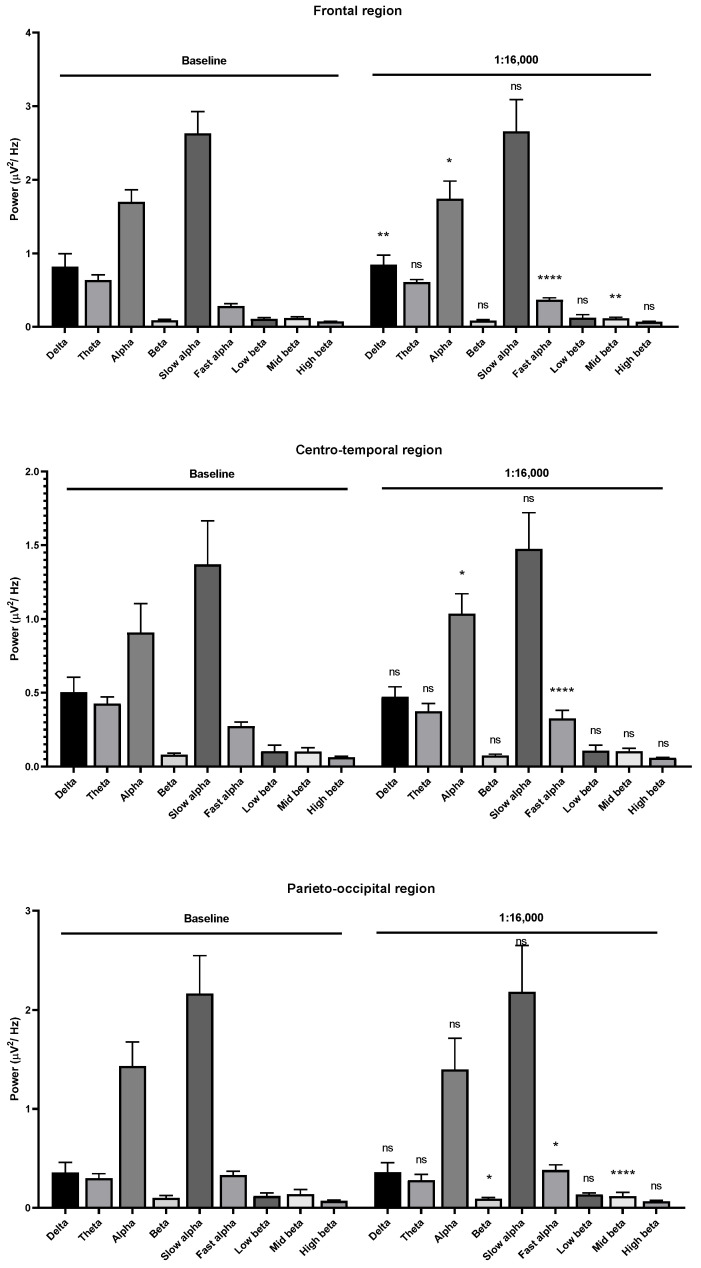
Effects of garlic oil inhalation at a 1:16,000 dilution on the delta, theta, alpha, beta, slow alpha, fast alpha, low beta, mid beta, and high beta wave powers: (**upper**) frontal region; (**middle**) centro-temporal region; (**lower**) parieto-occipital region. *p* value: not significant (ns) ≥ 0.05, * < 0.05, ** < 0.01, and **** < 0.0001.

**Figure 8:**
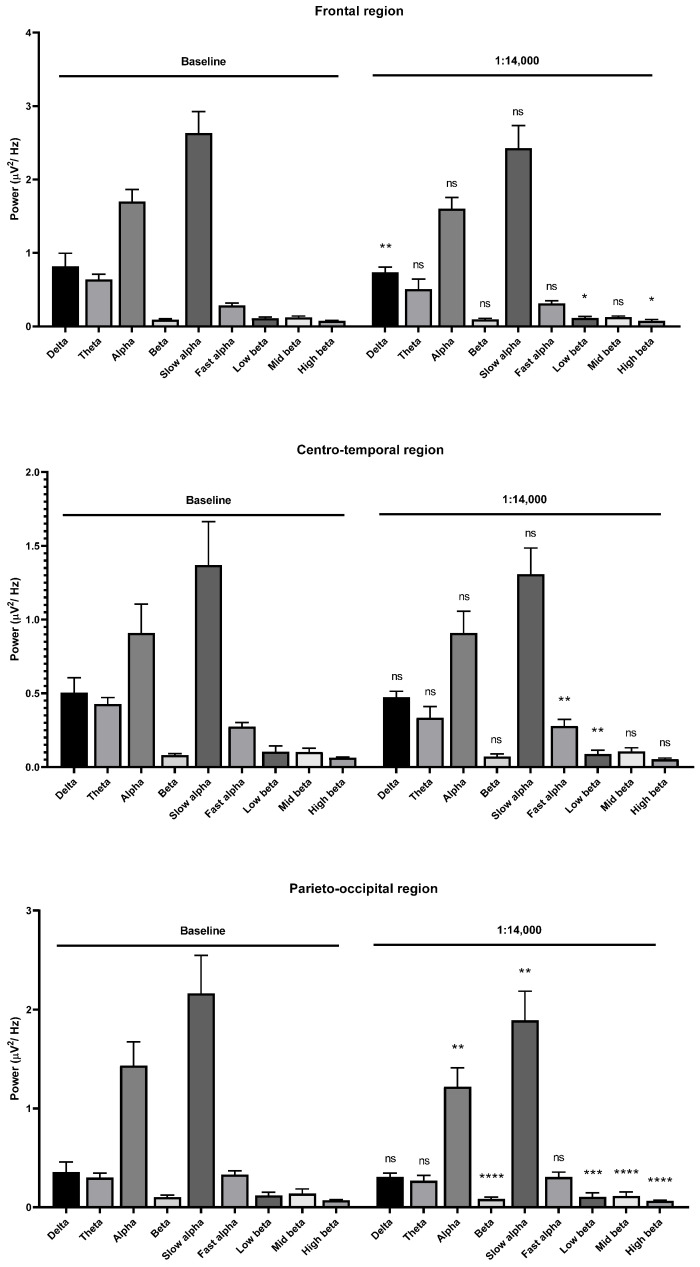
Effects of garlic oil inhalation at a 1:14,000 dilution on the delta, theta, alpha, beta, slow alpha, fast alpha, low beta, mid beta, and high beta wave powers: (**upper**) frontal region; (**middle**) centro-temporal region; (**lower**) parieto-occipital region. *p* value: not significant (ns) ≥ 0.05, * < 0.05, ** < 0.01, *** < 0.001, and **** < 0.0001.

Theta wave, alpha wave, and beta wave powers were decreased with the 1:14,000 and 1:12,000 dilutions. The alpha wave and beta wave powers showed no difference at the 1:10,000 dilution. The fast alpha wave power was increased in the frontal region during 1:16,000 oil dilution inhalation (Figure 7). The mid beta wave power was decreased. The fast alpha wave power was also increased in the centro-temporal and parieto-occipital regions. The mid beta wave power was decreased in the parieto-occipital region. Slight changes of brainwave power were observed in the frontal and centro-temporal regions during 1:14,000 dilution inhalation (Figure 8).

Low and high beta wave powers were increased in the frontal region, and the fast beta wave power was also increased in the centro-temporal region. The low beta wave power was decreased in the centrotemporal region. The parieto-occipital region showed clear changes during the 1:14,000 dilution oil inhalation. The alpha and beta wave powers were decreased. The slow alpha wave component was decreased, and all beta wave components were decreased in the parieto-occipital region. Interestingly, stronger garlic oil dilutions showed different alterations. The alpha wave power, especially the slow alpha wave power, was decreased in the frontal region during the 1:12,000 oil inhalation.

**Figure 9:**
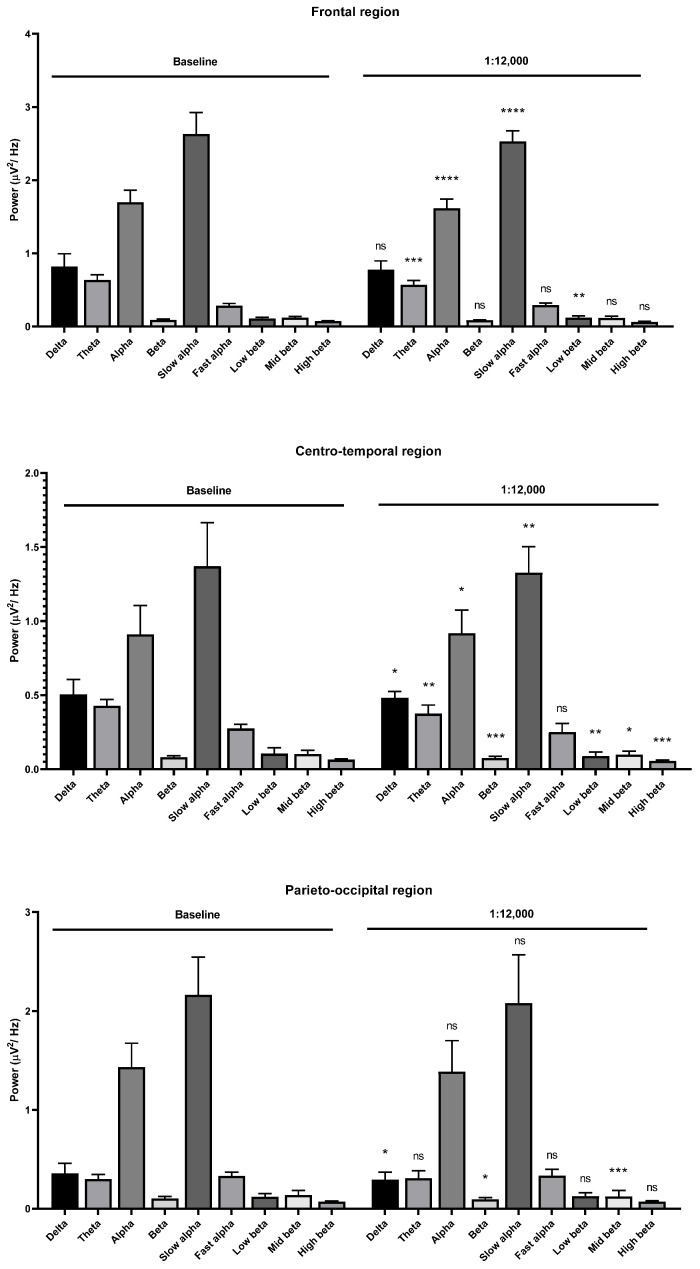
Effects of garlic oil inhalation at a 1:12,000 dilution on the delta, theta, alpha, beta, slow alpha, fast alpha, low beta, mid beta, and high beta wave powers: (**upper**) frontal region; (**middle**) centro-temporal region; (**lower**) parieto-occipital region. *p* value: not significant (ns) ≥ 0.05, * < 0.05, ** < 0.01, *** < 0.001, and **** < 0.0001.

**Figure 10:**
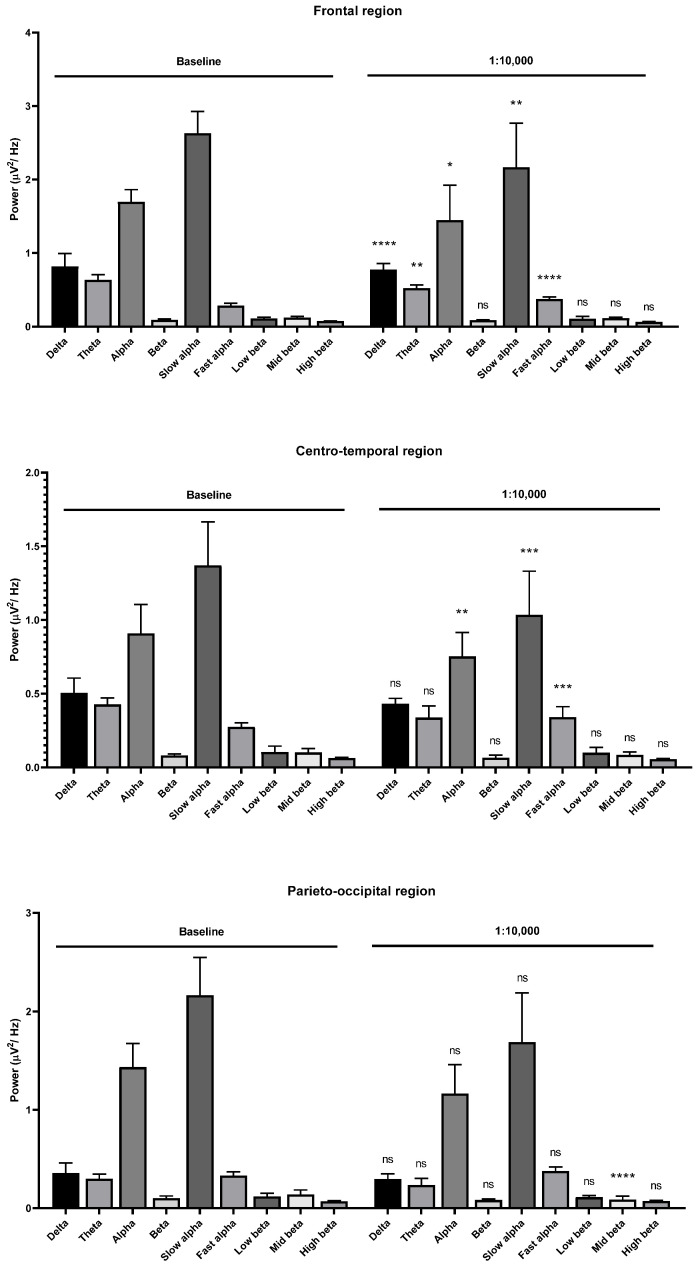
Effects of garlic oil inhalation at a 1:10,000 dilution on the delta, theta, alpha, beta, slow alpha, fast alpha, low beta, mid beta, and high beta wave powers: (**upper**) frontal region; (**middle**) centro-temporal region; (**lower**) parieto-occipital region. *p* value: not significant (ns) ≥ 0.05, * < 0.05, ** < 0.01, *** < 0.001, and **** < 0.0001.

The low beta wave power was slightly increased in the frontal region. The alpha and beta wave powers were decreased in the centro-temporal regions. The slow alpha, low beta, mid beta, and high beta wave powers were decreased. Only the mid beta wave power was decreased in the parieto-occipital region (Figure 9). During the 1:10,000 oil inhalation, the alpha wave power was decreased in both the slow and fast alpha components in the frontal region. The same result was observed in the centro-parietal region. The mid beta wave power was only decreased at parieto-occipital region (Figure 10).

**Figure 11:**
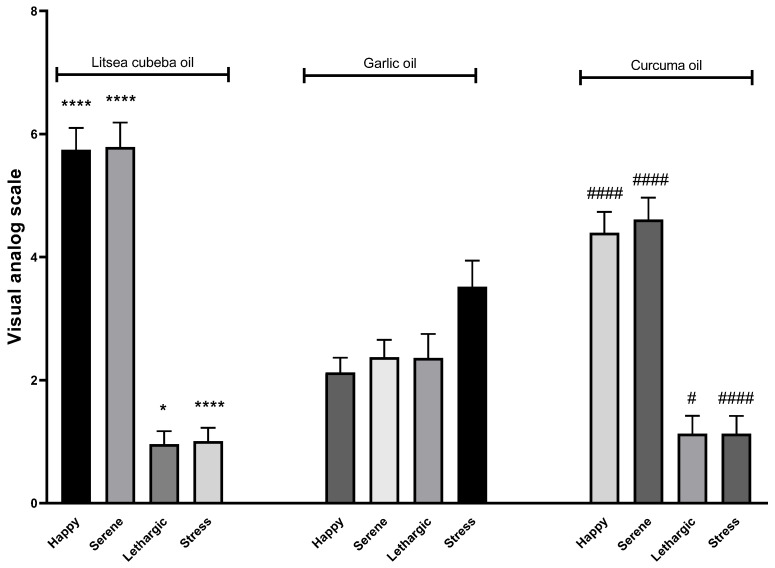
Effects of *Litsea cubeba*, garlic, and *Curcuma longa* oil inhalations on positive and negative moods; happy, serene, lethargic, and stress. *p* value compared *Litsea cubeba* oil with garlic oil: ns ≥ 0.05, * < 0.05, and **** < 0.0001. *p* value compared *Curcuma longa* oil with garlic oil: not significant (ns) ≥ 0.05, # < 0.05, and #### < 0.0001.

For mood evaluation, we chose three oils—*Litsea cubeba* oil, garlic oil, and *Curcuma longa* oil. Happy, serenity, lethargic, and stress emotions were assessed for each dilution. The average values of each oil perception are presented in Figure 11. *Litsea cubeba* oil and *Curcuma longa* oil showed more scores of positive moods. Happy and serene feelings with the *Litsea cubeba* and *Curcuma longa* oils were higher than with garlic oil inhalation. However, garlic oil inhalation showed more lethargy and stress feelings than with *Litsea cubeba* oil and *Curcuma longa* oil inhalations.

## 3. Discussion

Several Thai herbs have been used for centuries as ingredients in famous Thai foods. Lemongrass, garlic, and *Curcuma longa* are among the herbs used in Thai curry paste. Essential oils are released during the food preparation steps. We used *Litsea cubeba* oil instead of lemon grass oil due to its industry-scale readiness. The main *Litsea cubeba* components are citral and limonene, which are the same as in lemongrass. Limonene is also found in bergamot, caraway, eucalyptus, juniper, lemon, orange, and spearmint. In this study, we found that *Litsea cubeba* oil reduced alpha and beta wave powers.

The lower dilution, the 1:1000 dilution, caused clear changes of the brainwave powers in the temporal region. The stronger dilutions altered the brainwaves in a diffuse manner. The inhalation of *Litsea cubeba* oil showed more positive moods over negative ones. Orange oil also showed the same results and showed a relaxant effect with a positive mood [16,17]. Melissa extract, which is rich in citral, showed a sedative effect during resting conditions [18]. The odorants bind with odorant receptors and then send signals to the primary olfactory cortex, anterior olfactory nucleus, olfactory tubercle, piriform cortex, amygdala, and rostral entorhinal cortex. The piriform cortex, a sensory-associative cortex, connects with several high-order centers of the cerebral cortex. This connectivity pattern links to brain networks regulating cognition, memory, emotion, and behavior [19]. In addition to the olfactory system, evidence indicated effects from essential oil components in the systemic blood circulation passing through the nasal or lung mucosa [20].

Alteration of brain activity can affect the brain functions, including cognitive performance and sleep modification. The sedative effect of essential oil inhalation was also confirmed in several settings [15,21,22]. The reduction in both beta and alpha wave power activities induces calm state, shorter sleep latency, or deep sleep promotion. Limonene showed effects on brain neurotransmitters. A study showed effects of limonene on the γ-aminobutyric acid A receptor, which is related with the arousal pathway [23]. Citral is a type of monoterpene that was confirmed in inhibitory neurotransmitter modification [24,25]. This pharmacological effect reduces neuronal excitability, which benefits anxiety, insomnia, convulsion, pain, and cognitive deficits.

Garlic oil inhalation showed different effects between lower and higher concentrations. The alpha wave power was increased at the 1:16,000 dilution, and the alpha and beta wave powers were decreased at the 1:14,000 and 1:12,000 dilutions. Garlic oil demonstrated the lowest threshold property compared with *Litsea cubeba* and *Curcuma longa* oils. The increase in alpha wave power can be observed in several essential oil, such as white champaka, eucalyptus, lavender, and Zizyphus jujuba oils [14,26,27,28]. This alteration is related to psychometric responses, a relaxing effect, an increase in attention and relaxation.

Higher concentrations showed a sedative effect with *Litsea cubeba* oil. A neuroprotective effect was tested in garlic, and it showed anti-acetylcholinesterase activities [29,30]. Inhibition of the L-type calcium channel was tested in diallyl disulphide, diallyl trisulfide, and allyl tetrasulfide [31]. Negative moods were stimulated by garlic oil compared with the others. The fast alpha wave power was increased in the frontal and temporal region in 1:16,000 oil inhalation. The parietal and occipital regions were clearly changed regarding the brainwave powers for the stronger odor at a 1:14,000 dilution. Garlic essential oil has antidepressant-like effects. Its organosulfur component increases serotonin and dopamine levels [8].

Chronic treatment in an animal study demonstrated increased brain-derived neurotrophic factor, cyclic adenosine monophosphate response element-binding protein, and protein kinase B expression, which finally modulated the monoamine neurotransmitter. In our study, we analyzed the effects of *Litsea cubeba* and garlic oil on brainwaves using regional analysis. Positive and negative moods were evaluated during *Litsea cubeba*, garlic, and *Curcuma longa* oil inhalations. More numbers of volunteers are needed to assure the result of this type of behavior. The experiment and analysis were carried out to investigate each essential oil. The final products of food still require evaluation regarding brain activity and other psychophysiological functions. Brain functions and the psychophysiological responses can be investigated in the future. The knowledge obtained from this study can add value to both products and raw materials on the market.

## 4. Materials and Methods

### 4.1. Subject

**Figure 12:**
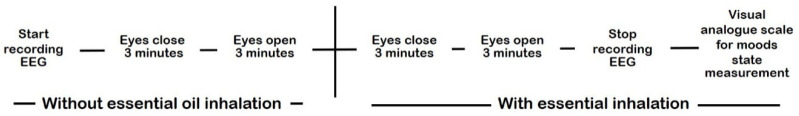
Experimental procedure divided into two trials: with and without essential oil inhalation. Three odors were applied for a volunteer with repeated protocols.

Thirty female healthy subjects were recruited in this study. The experimental protocol was approved by the Human Research Ethics Committee of Walailak University. The approval numbers are WUEC-19-140-01 and WUEC-21-038-01. The inclusion criteria were female participants without the menstrual period on the experimental day, normal smell performance, and without severe neurological injury history. All subjects had adequate sleep prior to the experimental day at approximately 6 h. Alcohol, smoking, tea and coffee were forbidden for a week before experiment. A smell test was evaluated using ten odorant stimuli. The included subjects were further assessed as the following experimental protocol in Figure 12. A motor task was also tested to include only right-handed persons. Demographic data were shown in Table 3. The method was slighted modified from Kim et al., 2018, and Koomhin et al., 2020 [14,32]. To replicate odor or fragrance application in the real-life situation, the odor was attached near volunteer nose. Adaptation process is not occurred with this method.

### 4.2. Essential Oil Preparations, GC–MS Analyses and Administrations

*Litsea cubeba*, garlic, and *Curcuma longa* oils extracted by steam distillation were obtained from Thai China Flavours & Fragrances Company Limited (Nonthaburi, Thailand). The oils were analyzed by a 6890/5973 GC–MS instrument (Agilent Technologies, Inc., Santa Clara, CA, USA) using two VF-WAXms columns (30 m × 250 µm, film thickness 0.25 μm). The oven temperature was held at 35 °C for 4 min and then was programmed to 200 °C at 10 °C/min for 5 min and then to 270 °C at 5 °C/min for 10 min; the injector and detector temperatures were set at 270 °C, and the ion source temperature was 150 °C; the helium carrier gas flow rate was 1 mL/min; the splitless injection was 75 mL/min for 0.5 min; the injection volume was 1 μL (Chromatograms provided in Supplementary Materials).

The compounds were identified by their retention indices and their mass spectra. The components of the oil were identified by comparison of their mass spectra fragmentation and computer matching using the Wiley 7n libraries (Database/ChemStation data system). The experimental protocol was adapted from Sowndhararajan et al. [33]. The subjects were asked to sit quietly in a recliner to limit movement of their head. The essential oil application was modified from other studies. Three dilutions were prepared for each oil using seventy percent ethanol as a carrier. The subjects were asked to open and close their eyes for three-minute intervals during the absence and presence of essential oils. The blinking was allowed to naturally occur during eyes open period. Fifty microliters of each different dilution of oil were dropped onto a filter paper. The filter paper with or without the essential oil was then attached to the subject’s nose. An electroencephalogram was recorded during the non-task resting state.

### 4.3. Electroencephalography

The electroencephalography (EEG) was recorded using a SynAmps RT 64-channel amplifier (Compumedics Limited, Victoria, Australia). The subjects wore an electrode cap, Quikcap, on their heads, and nineteen channels were loaded with Quikgel to reduce the impedance. FP1, FP2, F3, F1, FZ, F4, F8, T7, C3, CZ, C6, T8, P7, P3, PZ, P4, P8, O1, and O2 electrodes were recorded with a sampling rate of 1 kHz. M1 and M2 were the reference electrodes. Five kΩ was accepted for the electrode impedance. The raw data during the eye-closed periods were then analyzed with a triplicate of a 10 s epoch size.

The raw data were processed, filtered, and artifact-reduced using Curry 7 software (Compumedics Limited, Victoria, Australia). Notch filter at 50 Hz was processed. High pass and low pass filters were set to 1 Hz and 30 Hz, respectively. Three 10 s epochs of the eyes closed period were chosen to be analyzed per testing condition. Eye blink artifacts were removed using electrooculographic electrode VEO as a probe. The time-domain data were then transformed into frequency-domain data using fast Fourier transform. Power spectral analysis was finally performed to clearly display the differences among frequencies. The total brain response was processed and displayed in a bar graph. For the regional analysis, electrodes were chosen and were divided into three categories consisting of the frontal region (FP1, FP2, F3, F1, FZ, F4, and F8), centro-temporal region (T7, C3, CZ, C6, and T8), and parieto-occipital region (P7, P3, PZ, P4, P8, O1, and O2).

### 4.4. Score of Moods State Response

Emotional words were modified from James Russel [34,35]. Four representative positive and negative moods were selected. Happy and serene were the positive moods. Lethargy and stress were the negative moods. The meanings of these English words were translated into Thai language. The explanation was instructed to the volunteers preventing meaning confusion. The visual analog scales for self-assessment of the mood state of the subjects were evaluated after the inhalation of essential oil. The internal mood was scored by the individual subject by marking on a 10 cm horizontal line. Three oils were tested for a volunteer. To minimize carryover effect, the oils were given in this concentration order from the lowest concentration to the highest concentration.

### 4.5. Data Analyses and Statistics

The EEG data are shown as the median with a 95% CI. The mood scores are reported as the mean ± the standard error of the mean. Analyses of variance were performed followed by the Wilcoxon matched-pairs signed rank test for non-parametric data. Statistical differences were considered at p-values less than 0.05. EEG brainwave powers were processed by power spectral analysis and frequency domain data of subbands, including slow alpha (8–11 Hz), fast alpha (11–13 Hz), low beta (13–15 Hz), mid beta (15–20 Hz), and high beta (20–30 Hz). Mood scores were tested using an unpaired t test. All the statistics and graph preparations were produced using GraphPad Prism 9 (San Diego, California, United States).

## 5. Conclusions

*Litsea cubeba* oil inhalation showed a sedative effect. Garlic increased the alpha wave power at a low dilution. Higher dilutions also showed a sedative effect. The frontal and temporal regions of the brain were responsible for the response to *Litsea cubeba* oil inhalation. The temporal, parietal, and occipital regions of the brain were altered during higher dilutions of garlic oil inhalation. *Litsea cubeba* and *Curcuma longa* oil showed better positive mood scores, while garlic oil showed more negative mood scores.

## Figures and Tables

**Table 1:** Chemical compositions of *Litsea cubeba* oil.

RT Min	% (Area)	Compound
4.99	0.07	α-Thujene
5.12	2.57	Pinene
5.38	0.46	Camphene
5.71	2.16	Sabinene
5.81	1.66	β-pinene
5.88	0.65	6-Methyl-5-hepten-2-one
5.92	1.73	β-Myrcene
5.98	0.12	2,3-dihydro-1,8-cineole
6.58	20.44	dl-Limonene
6.65	2.12	1,8-Cineole
7.02	0.11	γ-Terpinene
7.44	0.10	α-Terinolene
7.63	1.24	l-Linalool
8.30	0.14	Citral
8.42	0.61	Citronella
8.83	1.15	Cyclohexanone,5-methyl-2-(1-methylethenyl)-, trans-
8.91	0.35	Terpinen-4-ol
9.12	0.49	α-Terpineol
9.50	0.59	Geranyl Alcohol
9.71	26.78	β-Citral
9.85	1.01	Linalool
10.12	33.36	α-Citral
11.64	0.09	α-Cubebene
12.25	0.93	Trans-Caryophyllene
13.21	0.13	Bicyclogermacrene
**Total**	**99.07**	

**Table 2:** Chemical compositions of garlic oil.

RT Min	% (Area)	Compound
2.34	3.19	Allyl methyl sulfide
3.88	0.29	1,2-Dithiolane
4.05	30.17	Diallyl sulfide
4.25	0.20	*n*-Propyl trans-1-propenyl sulfide
4.87	3.05	Allyl methyl disulfide
5.70	0.25	Dimethyl trisulfide
7.39	24.40	Diallyl disulphide
7.59	0.30	Allyl propyl disulfide
8.30	5.00	Trisulfide, methyl 2-propenyl
9.09	0.13	3-Vinyl-4*H*-1,2-dithiin
9.47	0.73	3-Vinyl-1,2-dithiocyclohex-5-ene
10.67	18.00	Diallyl trisulfide
10.83	0.31	Isobutyl isothiocyanate
11.78	0.91	Dimethyl tetrasulphide
12.57	0.23	Dimethylthioformamide
13.52	0.82	2-(2-thia-4-pentenyl)-1-thia-cyclohex-5-ene
13.81	5.28	Diallyl tetrasulphide
14.28	0.15	3*H*-1,2,4-Triazole-3-thione, 4,5-dihydro-4-methyl-
14.39	0.46	Isobutyl isothiocyanate
21.29	0.34	Octasulfur
**Total**	**94.21**	

**Table 3:** Baseline characteristics for brainwave studies. Data are shown as the mean ± standard deviation.

Essential Oil	n	Age (Years)	Weight (kg)	Height (cm)	Body Mass Index (kg/m^2^)	Smell Test Score (10)	Handedness
Garlic	10	21.60 ± 1.35	55.72 ± 9.59	159.10 ± 7.75	22.04 ± 3.77	7.90 ± 0.88	Right
*Litsea cubeba*	10	21.80 ± 1.75	50.89 ± 6.46	158.45 ± 6.20	20.44 ± 2.83	8.50 ± 0.97	Right
*Curcuma longa*	10	21.80 ± 0.42	53.50 ± 11.91	157.00 ± 3.83	21.64 ± 4.48	9.30 ± 0.82	Right

## Data Availability

The presented data are available on reasonable request from the corresponding author.

## Supplementary Materials

The following are available online, Figure S1: Chromatogram of *Litsea cubeba* oil analysis by GC-MS, Figure S2: Chromatogram of garlic oil analysis by GC-MS, Figure S3: Chromatogram of *Curcuma longa* oil analysis by GC-MS, Table S1: Chemical composition of *Curcuma longa* oil.

## Sample Availability

Samples of the compounds are not available from the authors.
