# Supplementary material for: Effects of Thai Local Ingredient Odorants, Litsea cubeba and Garlic Essential Oils, on Brainwaves and Moods
Source: Molecules. 2021 May 15;26(10):2939. doi: 10.3390/molecules26102939 (PMC8156645; doi:10.3390/molecules26102939)
Supplement: Supplementary file 1 [file molecules-26-02939-s001.zip › molecules-1185886-supplementary.pdf]

molecules

Supplementary Materials

Effects of Thai Local Ingredient Odorants, *Litsea cubeba* and Garlic Essential Oils, on Brainwaves and Moods

Apsorn Sattayakhom [1,2], Sumethee Songsamoe [2,3], Gorawit Yusakul [4], Kosin Kalarat [5], Narumol Matan [2,3] and Phanit Koomhin [2,6*]

1 School of Allied Health Sciences, Walailak University, Nakhonsithammarat, Thailand; apsorn.sa@wu.ac.th
2 Center of Excellence in Innovation on Essential oil, Walailak University, Nakhonsithammarat, Thailand; apsorn.sa@wu.ac.th (A.S.); sumethee.so@wu.ac.th (S.S.); nnarumol@wu.ac.th (N.M.); phanit.ko@mail.wu.ac.th (P.K.)
3 School of Agricultural Technology, Walailak University, Nakhonsithammarat, Thailand; sumethee.so@wu.ac.th (S.S.); nnarumol@wu.ac.th (N.M.)
4 School of Pharmacy, Walailak University, Nakhonsithammarat, Thailand; gorawit.yu@wu.ac.th
5 School of Informatics, Walailak University, Nakhonsithammarat, Thailand; kosin.ka@wu.ac.th
6 School of Medicine, Walailak University, Nakhonsithammarat, Thailand; phanit.ko@mail.wu.ac.th
* Correspondence: phanit.ko@mail.wu.ac.th; Tel.: +66-95295-0550

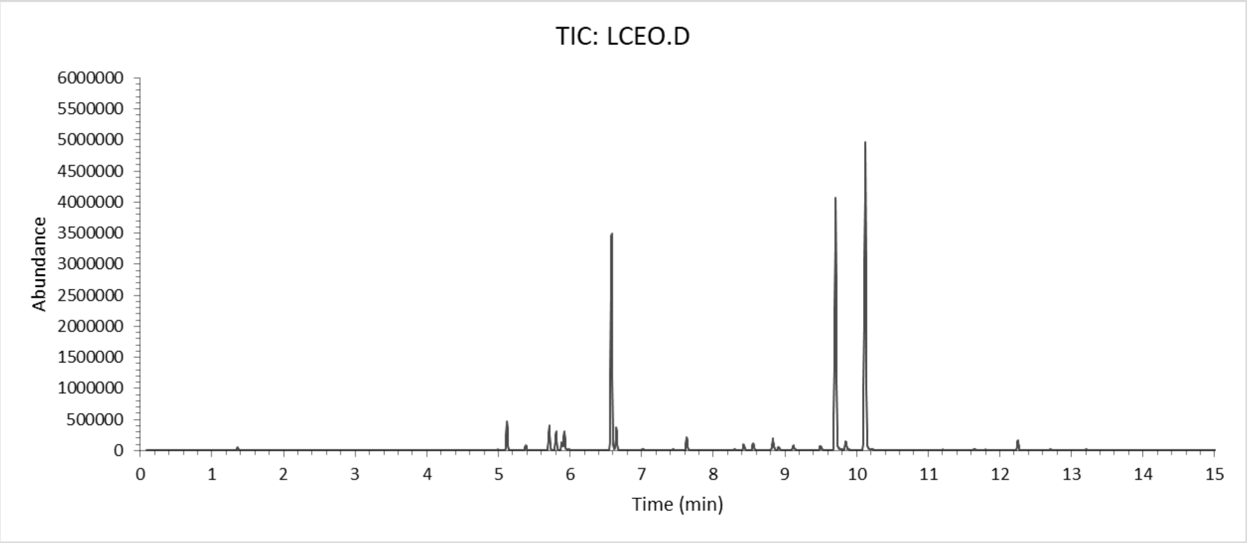

Figure S1. Chromatogram of *Litsea cubeba* oil analysis by GC-MS.

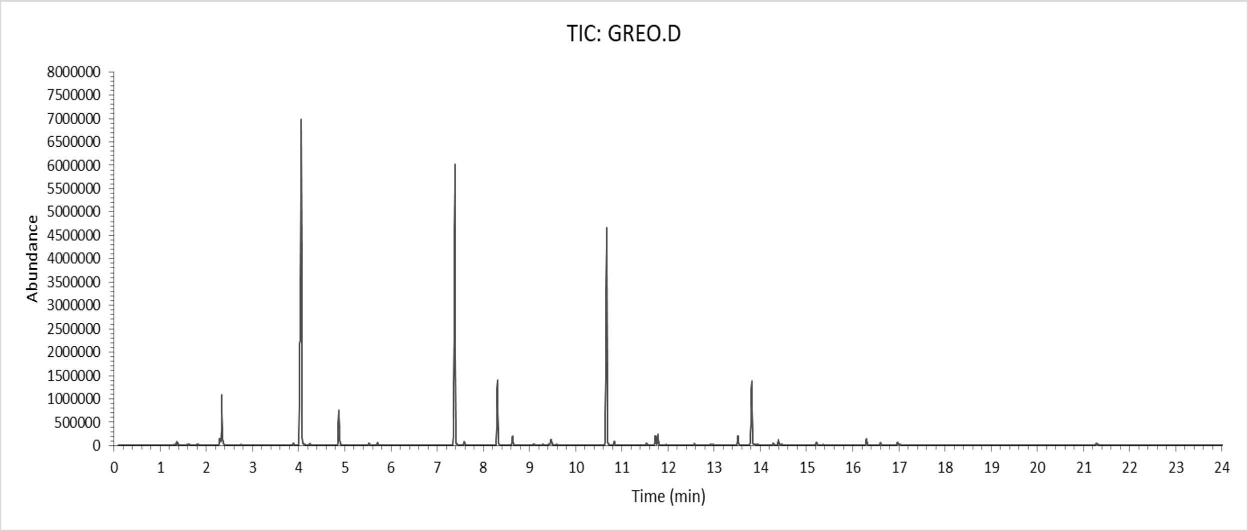

Figure S2. Chromatogram of garlic oil analysis by GC-MS.

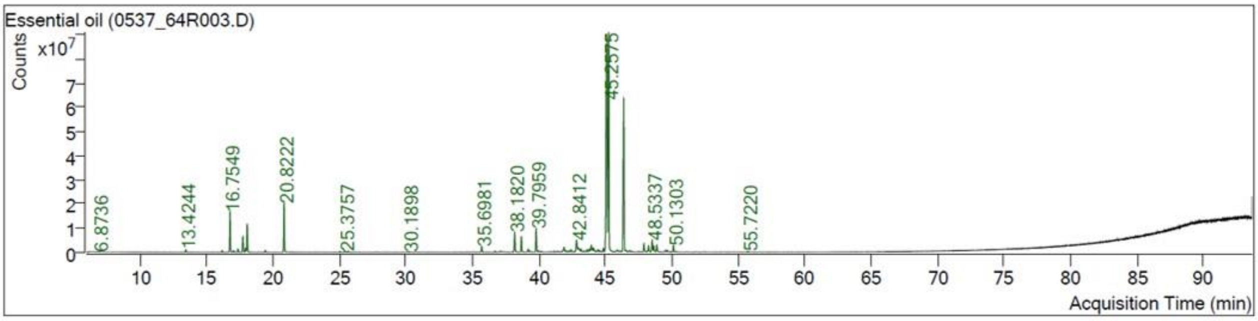

Figure S3. Chromatogram of *Curcuma longa* oil analysis by GC-MS.

Table S1. Chemical composition of *Curcuma longa* oil.

RT Min	% (Area)	Compound
16.7549	3.51	alpha-Phellandrene
17.7298	1.39	beta-Cymene
18.0479	2.53	1,8-Cineole
20.8222	4.57	alpha-Terpinene
35.6981	0.54	Caryophyllene
38.182	1.71	alpha-Curcumene
38.6826	1.34	Zingiberene
39.7959	1.99	beta-Sesquiphellandrene
42.8412	1.41	1-(3-Cyclopentylpropyl)-2,4-dimethylbenzene
45.1021	28.91	ar-Turmerone

45.2575	24.72	Tumerone
46.3855	15.75	beta-Turmerone
47.9115	0.76	(6R,7R)-Bisabolone
48.2633	0.59	(Z)-gamma-Atlantone
48.5337	1.11	5A-Methyl-3,8-Dimethylene-2-Oxododecahydrooxireno[2',3':6,7]Naphtho[1,2-B]Furan-6-YL 2-Methyl-2-Butenoate
48.6503	0.51	Cyclohexanol, 5-methyl-2-(1-methyl-1-phenylethyl)-
48.8796	0.66	(E)-Atlantone
50.1303	0.56	Benzenemethanol, 4-methyl-alpha-(1-methyl-2-propenyl)-, (R*,R*)-
Total	92.56	
